# The Unmarried Mother and Her Child

**Published:** 1920-12-04

**Authors:** Lettice Fisher, H. A. L. Fisher

**Affiliations:** Chairman of the Executive Committee. Evelyn House, 62 Oxford Street, W. 1; Chairman of the Executive Committee. Evelyn House, 62 Oxford Street, W. 1


### The Unmarried Mother and her Child.

Sir,—In view of recent discussion and correspondence, and in order to prevent any possible misunderstanding, the Executive Committee of the National Council for the Unmarried Mother and her Child wishes to state that the objects of the Council are the better care of the unmarried mother and her child, also the maintenance of a high standard of morality, and consequently the diminution of unmarried motherhood.—Yours faithfully,

Lettice Fisher (Mrs. H. A. L. Fisher), Chairman of the Executive Committee.

Evelyn House, 62 Oxford Street, W. 1,
November 23, 1920.

**We have received other letters which are unavoidably held over until next week.—Ed. "The Hospital."**

